# Mildly elevated serum bilirubin and its correlations with lipid levels among male patients undergoing health checkups

**DOI:** 10.1186/s12944-023-01979-w

**Published:** 2023-12-04

**Authors:** Xin Bai, Jing Qiao, Hong Zhang

**Affiliations:** 1 Department of Internal Medicine, Ningxia People’s Armed Police Corps Hospital, 895 Qinghe South Street, Yinchuan, 750001 Ningxia Hui Autonomous Region China; 2 Department of Outpatient, Ningxia People’s Armed Police Corps Hospital, 895 Qinghe South Street, Yinchuan, 750001 Ningxia Hui Autonomous Region China

**Keywords:** Direct bilirubin (DBil), Triglyceride (TG), Indirect bilirubin (IBil), Total cholesterol (TC), Total bilirubin (TBil)

## Abstract

**Background:**

Bilirubin’s ability to lower lipid levels was confirmed by several studies, but those studies mainly focused on total bilirubin (TBil). The present study aimed to elucidate the correlations of the two subtypes of bilirubin with lipid levels.

**Methods:**

A total of 1732 male patients undergoing health checkups were categorized into three groups according to the levels of direct bilirubin (DBil) and indirect bilirubin (IBil). The differences in medical characteristics among the three groups were analysed.

**Results:**

Subjects in the elevated DBil group had the lowest serum alanine aminotransferase (ALT), total cholesterol (TC), blood urea nitrogen (BUN), γ-glutamyl transpeptidase (γ-GT), fasting blood glucose (FBG), haemoglobin (HGB), and triglyceride (TG) levels in contrast to the other groups (*P* < 0.01), while subjects in the elevated IBil group had the highest ALT, γ-GT, BUN, serum creatinine (SCR), HGB, TC, and TG levels among the three groups (*P* < 0.01). DBil levels exhibited a significant negative correlation with TC (*r* = -0.777,* P* < 0.01) and TG (*r* = -0.397,* P* < 0.01) levels, while IBil levels exhibited a significant positive correlation with TC (*r* = 0.790,* P* < 0.01) and TG (*r* = 0.302,* P* < 0.01) levels. The frequencies of abnormal TC, TG, HGB and BUN levels were the lowest in the elevated DBil group, while the levels of these four variables were the highest in the elevated IBil group. Mildly elevated DBil levels were related to lower TG (OR = 0.112, 95% CI = 0.027–0.458) and TC (OR = 0.097, 95% CI = 0.013–0.700), and mildly elevated IBil levels were connected with increased TC (OR = 3.436, 95% CI = 2.398–4.924) and TG (OR = 1.636, 95% CI = 1.163–2.303). DBil was an independent protective factor against increased TC (OR = 0.702, 95% CI = 0.602–0.817, *P* < 0.01) and TG (OR = 0.632, 95% CI = 0.541–0.739, *P* < 0.01) levels, and IBil was an independent risk factors for increased TC (OR = 1.251, 95% CI = 1.176–1.331, *P* < 0.01).

**Conclusions:**

DBil was an independent protective factor against high TC and TG levels. IBil was an independent risk factors for elevated TC levels. The prognostic value of IBil levels warrants further attention.

## Introduction

Lipids are a group of organic compounds that have various physiological functions [1, 2]. Dyslipidaemia, often manifesting as elevated triglyceride (TG), low-density lipoprotein (LDL) cholesterol, and total cholesterol (TC) levels and diminished high-density lipoprotein (HDL) cholesterol levels [3], has been shown to contribute to the progression of cardiovascular diseases (CVDs), diabetes and obesity [4, 5].

Bilirubin, one product of haemoglobin (HGB) metabolism, was once regarded as a harmful waste product that has no biological activities. Recently, a growing body of research has shown that bilirubin plays a potential role in different physiological and metabolic activities as a “yellow hormone” [6]. Research on bilirubin’s physiological activity was first conducted in individuals with Gilbert’s syndrome (GS), a disease that causes serum bilirubin levels to be mildly elevated due to the hypoactivity of glucuronosyl transferase [7]. In this patient group, the prevalence of ischaemic heart disease exhibited a statistically significant decrease compared to the control group [8, 9]. Several recent studies have already illustrated the function of mildly elevated bilirubin in CVDs and metabolic diseases [10–14]. Total bilirubin (TBil) is composed of direct bilirubin (DBil) and indirect bilirubin (IBil). Some studies have reported the association between TBil and lipid levels. Oda et al. reported that a decrease in serum TBil could be a predictor of high LDL cholesterol [15]. In another Asian group, Choi et al. reported the same result that high bilirubin was negatively associated with high TC levels [16]. Without classification of DBil and IBil levels, these studies might fail to differentiate the actual effects of DBil and IBil on lipid levels. Relevant reports that mainly emphasize the connection between these two subtypes of bilirubin and lipid levels remain scarce. Therefore, the present study investigated the connection between these two kinds of bilirubin and lipid levels in local male patients undergoing health checkups.

## Methods

### Study subjects

**Fig. 1:**
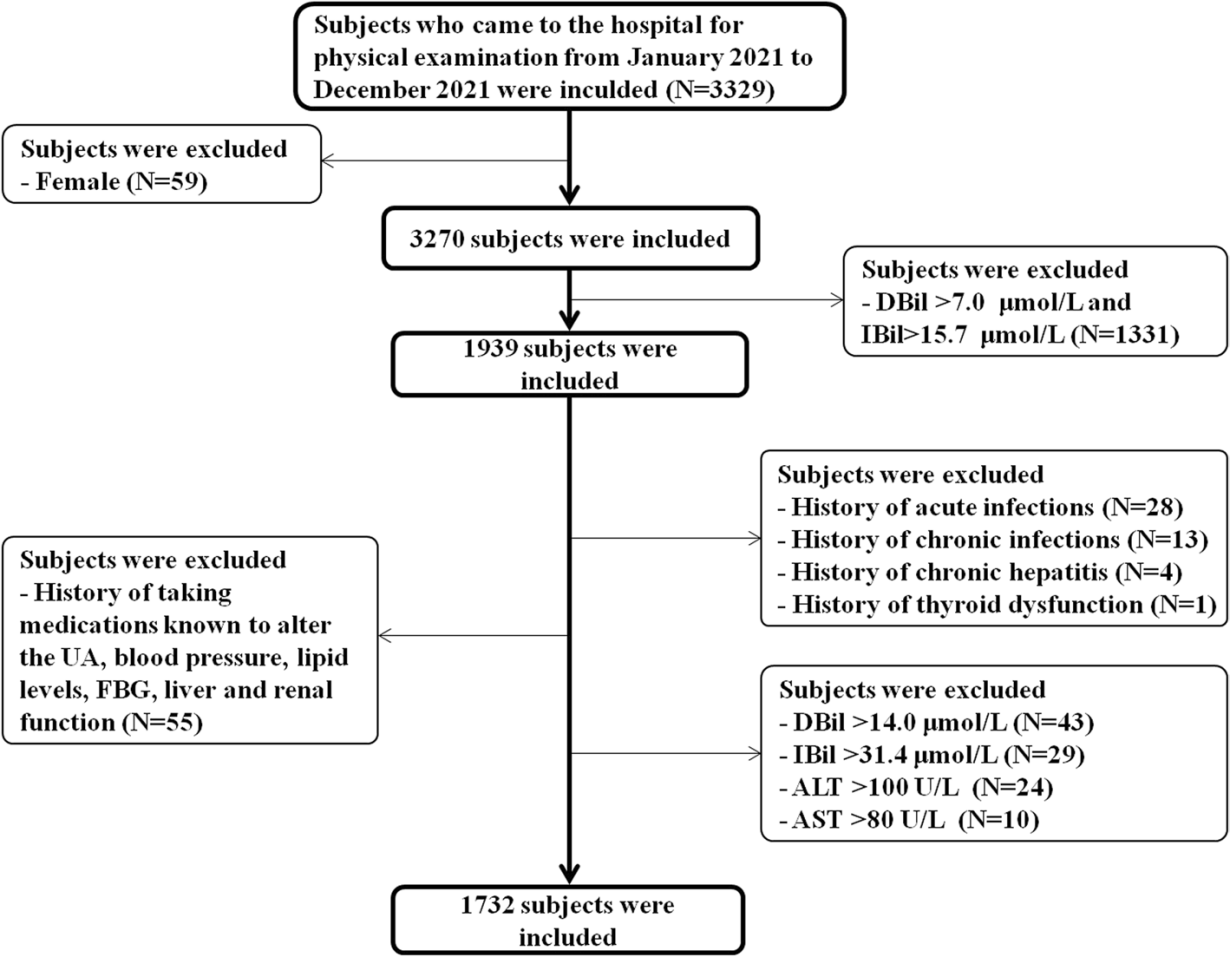
Flowchart of inclusion and exclusion

All subjects were healthy men aged between 19 and 49 years who came to the hospital for physical examinations from January 2021 to December 2021 (Fig. 1). Individuals who met the following conditions were excluded from the investigation: 1) female sex; 2) both elevated DBil (> 7.0 μmol/L) and elevated IBil (> 15.7 μmol/) levels; 3) a history of CVDs, haematological disease, acute or chronic infections, chronic hepatitis/cirrhosis, thyroid dysfunction, acute or chronic kidney insufficiency, anaemia, malignancy, or biliary obstruction disease; 4) a history of taking any medications known to alter uric acid (UA), blood pressure, lipid, fasting blood glucose (FBG) levels, liver and renal function; and 5) serum DBil, IBil, alanine aminotransferase (ALT) and aspartate aminotransferase (AST) levels greater than double the upper limit of normal (DBil level > 14 μmol/L, IBil level > 31.4 μmol/L, ALT level > 100 U/L, AST level > 80 U/L). Ultimately, the investigation included a total of 1732 individuals.

### Clinical and laboratory analysis

Trained medical workers assessed the systolic and diastolic blood pressure (SBP and DBP), in addition to the height and weight of all participants. The calculation of an individual’s body mass index (BMI) involved dividing their weight by the square of their height (kg/m2). In the morning, after 10 h of fasting, venous blood was drawn from all subjects. The blood samples were centrifuged at a force of 3000 × g for 15 min while being maintained at room temperature. A fully automatic biochemical analyser (Abbott Laboratories, Chicago, IL, USA) was utilized to detect TBil, AST, ALT, IBil, blood urea nitrogen (BUN), DBil, alkaline phosphatase (ALP), serum creatinine (SCR), UA, γ-glutamyl transpeptidase (γ-GT), FBG, HGB, TC, and TG levels.

### Groups and cases of abnormal variable definitions

Based on the levels of serum DBil and IBil, all participants were categorized into three groups: Group 1: 1177 subjects with standard DBil (< 7.0 μmol/L) and IBil (< 15.7 μmol/L) levels; Group 2: 170 subjects with elevated DBil (7.0–14.0 μmol/L) and normal IBil (< 15.7 μmol/L) levels; and Group 3: 385 subjects with elevated IBil (15.7–31.4 μmol/L) and normal DBil (< 7.0 μmol/L) levels. Laboratory variables that were above the upper-limit of normal were considered abnormal (ALT level > 50 U/L, TC level > 5.18 mmol/L, AST level > 40 U/L, γ-GT level > 60 U/L, ALP level > 125 U/L, BUN level > 8 mmol/L, SCR level > 115 μmol/L, UA level > 428 μmol/L, FBG level > 6.1 mmol/L, HGB level > 160 g/L, TG level > 1.7 mmol/L).

### Statistical analysis

Continuous variables with a normal distribution are represented as the means ± standard deviations (SDs), while variables that did not adhere to the normal distribution are represented as medians (25% quartile to 75% quartile); frequencies are used to represent categorical variables. Either one-way ANOVA or the Kruskal‒Wallis H test was utilized to evaluate variations among the three groups. For the examination of categorical variables, the investigation performed either the chi-square test or Fisher’s exact test. Simultaneously, the odds ratio (OR) and 95% confidence interval (95% CI) were computed. Spearman correlation and partial correlation analyses were conducted to assess the associations between bilirubin and lipid levels. Logistic analyses were performed to elucidate the independent factors for TC and TG levels. All statistical evaluations conducted in the investigation were two-tailed, and a significance level of 0.05 was considered indicative of statistical significance. The statistical analysis was conducted utilizing SPSS version 21.0, developed by SPSS Inc. in Chicago, IL, USA.

## Results

### Clinical variables in different groups

Table 1 presents the clinical variables of the subjects, and they were compared among the different groups. Except for current smoking, current drinking and AST levels, all variables, including SBP, DBP, ALT, BMI, ALP, γ-GT, BUN, SCR, UA, FBG, HGB, TC, and TG levels, exhibited significant variations among all groups (*P* < 0.05). Subjects in Group 2 had significantly decreased ALT, γ-GT, BUN, FBG, HGB, TC, and TG levels compared to the other groups (*P* < 0.01). Subjects in Group 3 had significantly increased ALT, γ-GT, BUN, SCR, HGB, TC, and TG levels compared to the other groups (*P* < 0.05). In Group 2, serum ALP and UA levels were significantly higher than those in the other groups (*P* < 0.05).

**Table 1:** Comparisons of clinical variables among the three groups based on the level of bilirubin

Parameters (unit)	Group 1 (1177)	Group 2 (170)	Group 3 (385)	*P* value
Age (years)	24(22—28)	23(21—24)	26(23—30)	0.000**
BMI (kg/m^2^)	22.34(20.81—24.18)	21.80(20.42—22.86)	22.65(21.22—24.42)	0.000**
SBP (mmHg)	113.04 ± 10.73	112.59 ± 11.00	114.92 ± 10.44	0.007**
DBP (mmHg)	67.10 ± 7.89	64.99 ± 7.20	68.18 ± 7.89	0.000**
Current smoking (%)	319(27.1%)	33(19.4%)	94(24.4%)	0.081
Current drinking (%)	185(15.7%)	23(13.5%)	47(12.2%)	0.217
HGB (g/L)	158.51 ± 11.20	156.13 ± 12.07	160.49 ± 10.62	0.000**
DBil (μmol/L)	4.70(4.00–5.40)	7.60(7.30–8.00)	6.40(5.80–6.80)	0.000**
IBil (μmol/L)	12.10(10.40–13.40)	14.70(13.60–15.30)	17.50(16.60–18.65)	0.000**
ALT (U/L)	17.40(14.00—23.20)	16.10(13.10—21.00)	19.30(15.10—26.40)	0.000**
AST (U/L)	21.90 ± 6.53	22.63 ± 6.76	22.71 ± 6.64	0.070
ALP (U/L)	86.50 ± 21.15	88.75 ± 20.94	84.28 ± 19.98	0.049*
γ-GT (U/L)	15.70(13.10—20.20)	13.85(11.80—18.05)	17.40(14.10—21.95)	0.000**
BUN (mmol/L)	5.48 ± 1.21	5.41 ± 1.15	5.77 ± 1.20	0.000**
SCR (μmol/L)	76.48 ± 8.81	77.31 ± 8.48	79.40 ± 9.89	0.000**
UA (μmol/L)	380.26 ± 76.90	394.50 ± 77.64	390.53 ± 75.22	0.012*
FBG (mmol/L)	4.91 ± 0.41	4.80 ± 0.39	4.91 ± 0.38	0.002**
TC (mmol/L)	3.90(3.49—4.35)	3.11(2.81—3.36)	4.50(4.14—4.93)	0.000**
TG (mmol/L)	0.85(0.66—1.19)	0.64(0.51—0.79)	1.00(0.76—1.37)	0.000**

Group 1: subjects with normal DBil and IBil levels; Group 2: subjects with elevated DBil and normal IBil levels; Group 3: subjects with elevated IBil and normal DBil levels

*BMI* Body mass index, *SBP* Systolic blood pressure, *DBP* Diastolic blood pressure, *HGB* Haemoglobin, *ALT* Alanine aminotransferase, *AST* aspartate aminotransferase, *ALP* Alkaline phosphatase, *γ-GT* γ-Glutamyl transpeptidase, *BUN* Blood urea nitrogen, *SCR* Serum creatinine, *UA* Uric acid, *FBG* Fasting blood glucose, *TC* Total cholesterol, *TG* Triglyceride, *DBil* Direct bilirubin, *IBil* Indirect bilirubin

^*^P < 0.05

^**^P < 0.01

### Correlations between bilirubin and lipid levels

Correlation analysis was conducted for DBil, IBil, and lipid levels, and Table 2 lists the results. Significant negative associations were found between DBil and TC and TG levels (*P* < 0.01). The correlation coefficient outcomes were -0.291 and -0.299, respectively. Significant positive associations were observed between IBil and TC (*r* = 0.360) and TG (*r* = 0.117) levels (*P* < 0.01). Additionally, a partial correlation analysis was performed, controlling for DBil and IBil levels as covariates (Table 3). The results showed a more obvious negative association between DBil and TC levels (*r* = -0.777, *P* < 0.01), while the association between DBil and TG levels did not change appreciably (*r* = -0.397, *P* < 0.01). More obvious positive correlations were found between IBil and TC (*r* = 0.790, *P* < 0.01) and TG (*r* = 0.302, *P* < 0.01) levels.

**Table 2:** Spearman correlation between bilirubin and TC and TG levels in all subjects

Parameters	DBil		IBil	
	*r*	*P* value	*r*	*P* value
TC	-0.291	0.000**	0.360	0.000**
TG	-0.299	0.000**	0.117	0.000**

*TC* Total cholesterol, *TG* Triglyceride, *DBil* Direct bilirubin, *IBil* Indirect bilirubin

^**^P < 0.01

**Table 3:** Partial correlation between bilirubin and TC and TG levels in all subjects

Parameters	DBil (IBil as covariates)		IBil (DBil as covariates)	
	*r*	*P* value	*r*	*P* value
TC	-0.777	0.000**	0.790	0.000**
TG	-0.397	0.000**	0.302	0.000**

*TC* Total cholesterol, *TG* Triglyceride, *DBil* Direct bilirubin, *IBil* Indirect bilirubin

^**^P < 0.01

### Analysis of patients with abnormal laboratory variables

The patients with abnormal laboratory variables were analysed in each group, and significant differences were found in four laboratory variables, including TC, TG, HGB and BUN levels (Table 4). The number of patients with abnormal TC, TG, HGB and BUN levels was the lowest in Group 2, and the frequencies were 0.6%, 1.2%, 34.7% and 0.6%, respectively. The number of patients with abnormal TC, TG, HGB and BUN levels was the highest in Group 3, and the frequencies were 17.4%, 14.8%, 52.5% and 4.9%, respectively. The variations among all groups were significant (*P* < 0.05). No significant variations of other abnormal laboratory variables were found among the three groups. Further analysis showed mildly elevated DBil levels were associated with decreased TC and TG levels, with ORs (95% CIs) of 0.097 (0.013–0.700) and 0.112 (0.027–0.458), respectively (*P* < 0.01), and mildly elevated IBil levels were associated with increased TC and TG levels, with ORs (95% CIs) of 3.436 (2.398–4.924) and 1.636 (1.163–2.303), respectively (*P* < 0.01) (Table 5). The results of univariate logistic analysis confirmed the association of bilirubin with TC and TG levels (Table 6). DBil levels were negatively associated with TC and TG levels, with ORs (95% CIs) of 0.685 (0.600–0.782) and 0.592 (0.552–0.671), respectively. IBil levels were positively associated with TC and TG levels, with ORs (95% CIs) of 1.298 (1.228–1.373) and 1.089 (1.037–1.143), respectively. After adjusting for age, BMI, smoking status, drinking status, SBP, DBP, HGB, ALT, AST, ALP, γ-GT, BUN, SCR, UA and FBG levels, DBil levels were an independent protective factor against high TC (OR = 0.702, 95% CI 0.602–0.817, *P* < 0.01) and TG (OR = 0.632, 95% CI 0.541–0.739, *P* < 0.01) levels, and IBil levels were an independent risk factor for high TC levels, with an OR (95% CI) of 1.251 (1,176–1.331).

**Table 4:** Comparisons of abnormal clinical variables among the three groups based on the level of bilirubin

Parameters(unit)	Group 1 (1177)	Group 2 (170)	Group 3 (385)	*P* value
ALT > 50 (U/L)	34(2.9%)	3(1.8%)	11(2.9%)	0.801
AST > 40 (U/L)	26(2.2%)	5(2.9%)	7(1.8%)	0.682
ALP > 125 (U/L)	60(5.1%)	10(5.9%)	15(3.9%)	0.525
γ-GT > 60 (U/L)	16(1.4%)	0(0.0%)	6(1.6%)	0.303
BUN > 8 (mmol/L)	39(3.3%)	1(0.6%)	19(4.9%)	0.031*
SCR > 115 (μmol/L)	0(0.0%)	0(0.0%)	1(0.3%)	0.320
UA > 428 (μmol/L)	284(24.1%)	45(26.5%)	95(24.7%)	0.796
FBG > 6.1 (mmol/L)	4(0.3%)	1(0.6%)	1(0.3%)	0.648
TC > 5.18 (mmol/L)	68(5.8%)	1(0.6%)	67(17.4%)	0.000**
TG > 1.7 (mmol/L)	113(9.6%)	2(1.2%)	57(14.8%)	0.000**
HGB > 160 (g/L)	517(43.9%)	59(34.7%)	202(52.5%)	0.000**

Group 1: subjects with normal DBil and IBil levels; Group 2: subjects with elevated DBil and normal IBil levels; Group 3: subjects with elevated IBil and normal DBil levels

*ALT* Alanine aminotransferase, *AST* Aspartate aminotransferase, *ALP* Alkaline phosphatase, *γ-GT* γ-Glutamyl transpeptidase, *BUN* Blood urea nitrogen, *SCR* Serum creatinine, *UA* Uric acid, *FBG* Fasting blood glucose, *TC* Total cholesterol, *TG* Triglyceride, *DBil* Direct bilirubin, *IBil* Indirect bilirubin, *HGB* Haemoglobin

^*^P < 0.05

^**^P < 0.01

**Table 5:** Odds ratios and 95% confidence intervals for the associations between bilirubin and TC and TG levels

Group	TC > 5.18(mmol/L)			TG > 1.7(mmol/L)		
	N (%)	OR (95% CI)	*P* value	N (%)	OR (95% CI)	*P* value
Group 1 (1177)	68(5.8%)	Reference		113(9.6%)	Reference	
Group 2 (170)	1(0.6%)	0.097(0.013–0.700)	0.004**	2(1.2%)	0.112(0.027–0.458)	0.000**
Group 3 (385)	67(17.4%)	3.436(2.398–4.924)	0.000**	57(14.8%)	1.636(1.163–2.303)	0.005**

Group 1: subjects with normal DBil and IBil levels; Group 2: subjects with elevated DBil and normal IBil levels; Group 3: subjects with elevated IBil and normal DBil levels

*TC* Total cholesterol, *TG* Triglyceride, *DBil* Direct bilirubin, *IBil* Indirect bilirubin, *N* Number, *OR* Odds ratio, *95% CI* Confidence interval

^**^P < 0.01

**Table 6:** Univariate and multivariate logistics analysis of associations between bilirubin and TC and TG levels

Parameters	TC				TG			
	Univariate analysis		Multivariate analysis		Univariate analysis		Multivariate analysis	
	OR (95% CI)	*P* value	OR (95% CI)	*P* value	OR (95% CI)	*P* value	OR (95% CI)	*P* value
DBil	0.685(0.600–0.782)	0.000**	0.702(0.602–0.817)	0.000**	0.592 (0.552–0.671)	0.000**	0.632 (0.541–0.739)	0.000**
IBil	1.298(1.228–1.373)	0.000**	1.251(1.176–1.331)	0.000**	1.089(1.037–1.143)	0.001**	1.045 (0.987–1.107)	0.131

*TC* Total cholesterol, *TG* Triglyceride, *DBil* Direct bilirubin, *IBil* Indirect bilirubin, *OR* Odds ratio, *95% CI* Confidence interval

^**^P<0.01

## Discussion

In the present study, male patients undergoing health checkups were classified into three groups depending on serum DBil and IBil levels. Significant differences, including those in ALT, SBP, DBP, BMI, ALP, BUN, UA, SCR, FBG, γ-GT, HGB, TC, and TG levels, were found among the three groups (*P* < 0.05). Correlation examination revealed that DBil levels were significantly negatively associated with TC and TG levels (*P* < 0.01), while IBil levels were significantly positively associated with the two lipid variables (*P* < 0.01). Further analysis showed that the frequencies of abnormal TC and TG levels were the lowest in the mildly elevated DBil group (Group 2), while the frequencies of abnormal TC and TG levels were the highest in the mildly elevated IBil group (Group 3).

Bilirubin comes from HGB released through the lysis of red blood cells, including DBil and IBil. IBil is water insoluble and cannot be excreted directly. After conjugation with glucuronic acid, IBil is converted to its water-soluble form known as DBil. Recently, many investigations have shown that bilirubin is a regulator with multiple biological functions [17, 18]. Slightly elevated bilirubin has been connected to a reduced incidence of cardiovascular events, diabetes, and metabolic syndrome [19–21]. In contrast to these studies, the subjects included in the current study were healthy patients, and the direct interaction between bilirubin levels and CVDs, diabetes, and metabolic syndrome cannot be derived from the present research, but some differences were observed. Laboratory variables, including TC, ALT, ALP, γ-GT, BUN, SCR, UA, FBG, HGB and TG levels, showed significant variation among the three groups (*P* < 0.05). The results from the current study showed that DBil and IBil may have different relationships with various biochemical markers.

Currently, the results of research on the association between bilirubin and lipid levels are still inconsistent. Fu et al. [22] reported that TBil and DBil levels were inversely related to TC and TG levels, while no significant correlation between IBil and the two lipid levels was observed. In the study by Seyed et al. [23], compared to men with low levels of bilirubin, those with mildly elevated bilirubin levels showed TG levels that were approximately 0.55 mmol/L lower. Oda et al. [24] found TBil levels were significantly correlated with the prevalence of increased TG in males in their cross-sectional study, while Zhang et al. [25] found no association between TBil and TG levels in Chinese individuals. Since TBil comprises DBil and IBil, the correlation among the serum bilirubin levels and the different levels of lipids should be assessed separately. In the current investigation, mildly elevated DBil levels were associated with decreased TC and TG levels (*P* < 0.01). Further investigation demonstrated that DBil levels were an independent protective factor against high TC and TG levels (*P* < 0.01). Bilirubin’s ability to lower lipid levels through direct interaction with peroxisome proliferator-activated receptor α (PPARα) has been confirmed by recent research [26, 27]. PPARα is mainly expressed in liver tissues [28], where DBil is mainly synthesized in hepatocytes. Under normal physiological conditions, IBil is powerfully bound to albumin for transportation and cannot be separated from albumin easily [29], while DBil might be easily separated from albumin. Thus, DBil had a hypolipidaemic effect on metabolism.

The present study revealed a positive correlation between IBil and TC levels for the first time, as no other similar reports of the positive correlation between IBil and TC levels are currently available. The possible explanation of IBil leading to dyslipidaemia is summarized below. Steyrer E et al. [30] reported that apolipoprotein D (Apo D) was an activator of lecithin-cholesterol acyltransferase (LCAT). LCAT is an enzyme that plays an important role in HDL metabolism and maturation [31, 32]. Clearance of excessive cholesterol termed “reverse cholesterol transport” is mediated by HDL [33]. The failure of reverse cholesterol transport will result in an increase in TC levels. In addition to strong binding to albumin, IBil has been confirmed to bind to Apo D for transportation in plasma [34]. A decrease in free Apo D levels may cause a decrease in LCAT activity, which in turn affects HDL maturation and clearance of excessive cholesterol. However, this is merely a conjecture that needs to be verified by further research.

### Study strengths and limitations

A strength that distinguishes the present study from previous studies is the classification of the study subjects. To investigate the real effects of different types of bilirubin and minimize confounding factors, a detailed group of all subjects was performed depending on DBil and IBil levels instead of TBil levels, and subjects with both elevated DBil and elevated IBil levels were excluded at the beginning of the study. Some limitations of the present research should be mentioned. First, all subjects included in the study were male, and the relationship between bilirubin and lipid levels in females could not be evaluated. Second, since the study was carried out in healthy patients undergoing checkups, there were no data on the values of serum HDL cholesterol and LDL cholesterol. Therefore, the correlation between bilirubin and these two atherogenic blood lipids could not be further studied. Finally, due to the lack of an additional validation cohort, the results of the current study need to be confirmed by an additional validation cohort study.

## Conclusions

This study categorized male patients undergoing health checkups into three groups based on the levels of serum IBil and DBil. Significant variations, including those in SBP, DBP, ALP, γ-GT, BMI, BUN, SCR, UA, ALT, FBG, HGB, TC, and TG levels, were found among the three groups. Mildly elevated DBil levels were related to lower TG and TC, and mildly elevated IBil levels were connected with increased TC and TG. DBil was an independent protective factor against high TC and TG levels. IBil was an independent risk factor for elevated TC levels. Although the exact mechanism of the positive relationship between IBil and TC levels is not fully understood, the prognostic value of IBil warrants further attention.

## Data Availability

The datasets used and analysed during the current study are available from the corresponding author on reasonable request.

## Abbreviations

TG: Triglyceride
LDL: Low-density lipoprotein
TC: Total cholesterol
HDL: High-density lipoprotein
CVDs: Cardiovascular diseases
HGB: Haemoglobin
GS: Gilbert’s syndrome
TBil: Total bilirubin
DBil: Direct bilirubin
IBil: Indirect bilirubin
UA: Uric acid
FBG: Fasting blood glucose
ALT: Alanine aminotransferase
AST: Aspartate aminotransferase
SBP: Systolic blood pressure
DBP: Diastolic blood pressure
BMI: Body mass index
BUN: Blood urea nitrogen
ALP: Alkaline phosphatase
SCR: Serum creatinine
γ-GT: γ-Glutamyl transpeptidase
SDs: Standard deviation
OR: Odds ratio
CI: Confidence interval
PPARα: Peroxisome proliferator-activated receptor α
Apo D: Apolipoprotein D
LCAT: Lecithin-cholesterol acyltransferase
